# Genome-wide detection of RNA editing events during the hair follicles cycle of Tianzhu white yak

**DOI:** 10.1186/s12864-022-08951-5

**Published:** 2022-10-31

**Authors:** Xuelan Zhou, Pengjia Bao, Xiaolan Zhang, Xian Guo, Chunnian Liang, Min Chu, Xiaoyun Wu, Ping Yan

**Affiliations:** 1 Key Laboratory of Animal Genetics and Breeding on Tibetan Plateau, Ministry of Agriculture and Rural Affairs, 730050 Lanzhou, P.R. China; 2 Key Laboratory of Yak Breeding Engineering Gansu Province, Lanzhou Institute of Husbandry and Pharmaceutical Sciences, Chinese Academy of Agricultural Sciences, 730050 Lanzhou, P.R. China

**Keywords:** RNA editing, Tianzhu white yak, Hair follicles cycle, Differential RNA editing sites

## Abstract

**Background:**

The hair coat is available for the yak to live in the harsh environment of the plateau. Besides, improving the hair production of yak is necessary for its textile industry development. Hair grows from hair follicles (HFs). The HFs undergo periodic growth after birth and are regulated by the complex gene regulatory network. However, the molecular mechanism of HFs regeneration in the Tianzhu white yak remains unclear. RNA editing is a post-transcriptional mechanism that regulates gene expression and produces new transcripts. Hence, we investigated the influence of the A-to-I RNA editing events on the HFs cycle of the Tianzhu white yak.

**Results:**

We finally identified 54,707 adenosine-to-inosine (A-to-I) RNA editing sites (RESs) from RNA sequencing data of the HFs cycle in the Tianzhu white yak. Annotation results showed RESs caused missense amino acid changes in 7 known genes. And 202 A-to-I editing sites altered 23 target genes of 140 microRNAs. A total of 1,722 differential RESs were identified during the HFs cycle of Tianzhu white yak. GO and KEGG enrichment analysis revealed several signaling pathways and GO terms involved skin development, hair growth, and HFs cycle. Such as genes with differential RNA editing levels were significantly enriched in the peroxisome, metabolic pathways, Notch signaling pathway, and PPAR signaling pathway. Besides, the editing sites in HFs development-related genes *FAS*, *APCDD1*, *WWOX*, *MPZL3*, *RUNX1*, *KANK2*, *DCN*, *DSC2*, *LEPR*, *HEPHL1*, and *PTK2B* were suggested as the potential RESs involving HFs development.

**Conclusion:**

This study investigated the global A-to-I RNA editing events during the HFs cycle of yak skin tissue and expanded the knowledge of A-to-I RNA editing on the HFs cycle. Furthermore, this study revealed that RNA editing-influenced genes may regulate the HFs cycle by participating in the HFs development-related pathways. The findings might provide new insight into the regulation of RNA editing in hair growth.

**Supplementary Information:**

The online version contains supplementary material available at 10.1186/s12864-022-08951-5.

## Background

Hair grows from hair follicles (HFs), and the HFs of animals could be divided into primer HFs and secondary HFs. After birth, the HFs distinctively undergo cyclical transformations, progressing through stages of anagen, catagen, and telogen [1]. Besides, the hair yield and quality of domestic animals are closely related to the diameter of hair fiber and the number of the HFs. Hair growth is a complex physiological and biological process, influenced by genetics, nutrition, and the environment [2–4]. Previous studies have reported that the HFs growth involves a complex process that is dependent on the coordinate regulation of several genes and signaling pathways, such as Wnt, mTOR, BMP, TGF-β, MAPK, Notch, etc. [5–9].

Tianzhu white yak (*Bos grunniens*) is an indigenous yak breed mainly distributed in the Gansu province of China, which is well known for its pure white coat. The well-proportioned coarse and fine hairs on the surface allow them to survive in the harsh environment of high altitude, characterized by strong wind, ultraviolet radiation, and low temperature [10]. The seasonal development of hair growth plays a useful role for them to adapt to the dynamically changing weather of the plateau. Besides, the white hair of yak is also used as the raw material in today’s textile industry because it is easily dyed with other colors. Previous studies mainly focused on hair color [11], hair structure [12], and hair production performance of Tianzhu white yak [13]. However, there are only have few studies about their hair growth. In recent years, transcriptional studies of the HFs cycle in Tianzhu white yak have been reported. Bao used RNA sequencing (RNA-Seq) to reveal the gene expression pattern of the seasonal hair cycle in Tianzhu white yak [14]. Zhang analyzed the expression pattern of lncRNAs during the HFs cycle of Tianzhu yak and explored the role of lncRNAs in the HFs cycle [15]. In addition, Bao analyzed the whole genome sequencing data between long-haired and normal-haired Tianzhu white yak to explore the hair length-related genes [16]. However, the molecular mechanism of HFs cyclical transformation in Tianzhu white yak still requires further investigation.

RNA editing is a widespread post-transcriptional process that can modify specific nucleotides in RNA sequences by site-specific deletion, insertion, and substitutions. RNA editing can result in differences between genomic sequences and the corresponding RNA sequences. As a result, RNA editing enhances the diversity of transcripts [17]. In mammals, adenosine-to-inosine (A-to-I) editing is the most common type catalyzed by adenosine deaminases acting on RNA (ADAR), and inosine is recognized as guanosine (G) in sequence data and also during RNA translation [18]. Most RNA editing sites (RESs) are located in non-coding regions, and a few editing sites are residing in coding regions and may alter the amino acid sequences of proteins. RNA editing also affects other transcriptional processes like alternative splicing and microRNA (miRNA) binding sites [19]. Previous studies have identified abundant RNA editing events, and the number and level of editing sites are varying among different development stages [20], cell lines [21], and environments [22]. It is important to explore the function of RNA editing response to different biological processes. Accordingly, numerous studies have reported that RNA editing is associated with brain development [23], spermatogenesis [24], cancer development, and metabolic disorders [25]. In light of this that millions of editing sites have been identified in domesticated animals, like sheep [26], bovines [27], chicken [28], and pig [29], etc. However, the study of RNA editing events has not been investigated in the yak genome.

RNA-sequencing could effectively analyze RNA editing events accompanied by bioinformatics development [30], which inspired us to explore the role of RNA editing in the HFs cycles of Tianzhu white yak. To explore the role of RNA editing events in hair growth. We used RNA-seq data to analyze A-to-I editing events of different periods during the HFs cycle in Tianzhu white yak. Then, we investigated the missense editing sites and explored the influence of RNA editing on miRNA target genes. Finally, the A-to-I sites with dynamically changed editing ratios were analyzed with the HFs cycle and investigated the key editing sites of the HFs cycle.

## Results

### Overview of RNA editing events in Tianzhu white yak

**Fig. 1:**
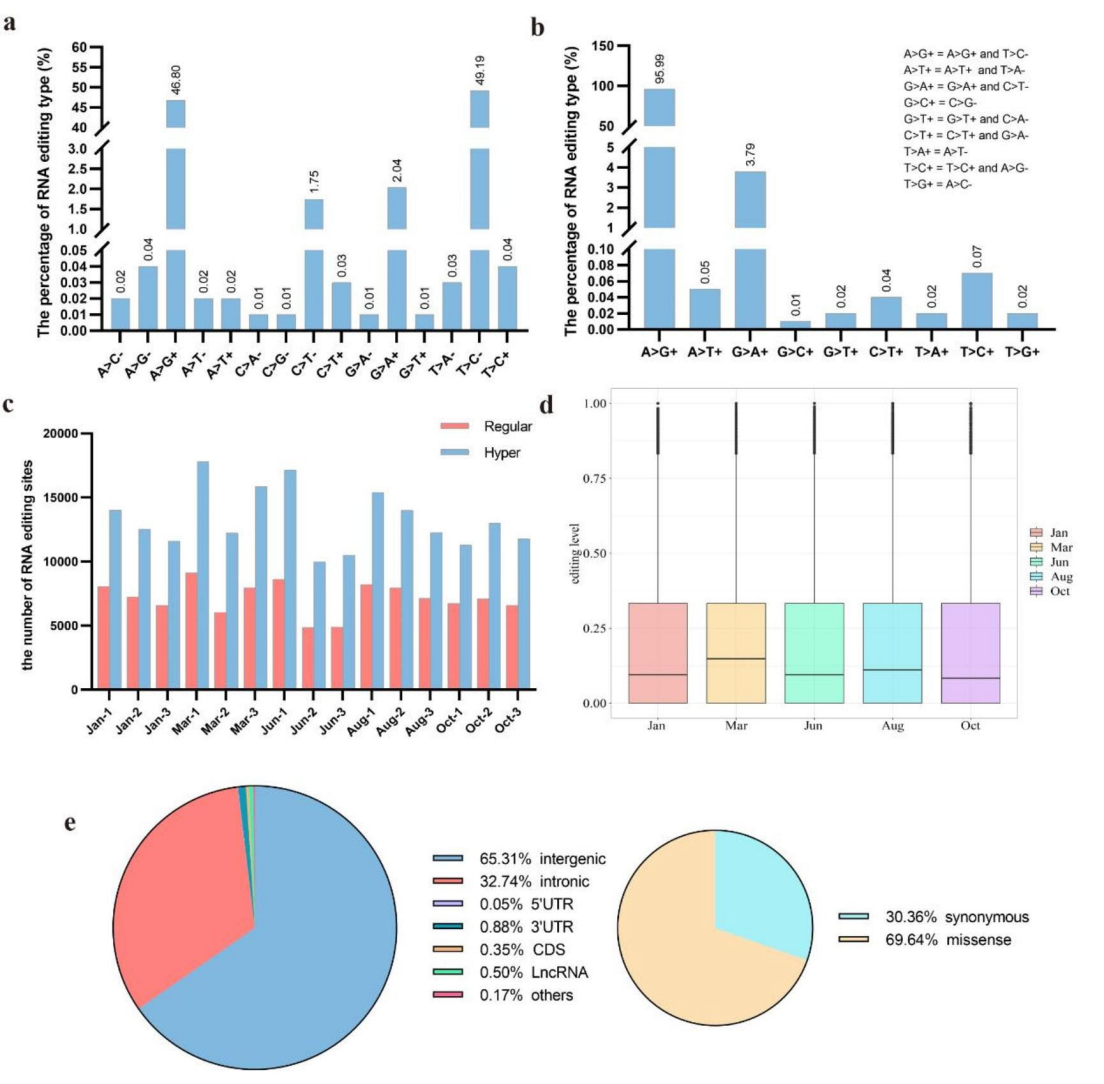
The overview of identified RNA editing events. (**a**, **b**) The statistical analysis of RNA editing types; (**c**) The number of the regular and hyper RNA editing sites; (**d**) The editing ratio distribution of all A-to-I editing events in different stages of the HFs cycle, editing level was calculated by the average editing level of three biological replicates of each stage; (**e**) Genomic distribution of RESs and the percentage of RESs caused missense variant in protein-coding region

In this study, we analyzed RNA-seq data of 15 skin samples from three vital periods across the HFs cycle of Tianzhu white yak to investigate the RNA editing events. We identified 56,994 RESs from 15 skin tissue samples. Because the data of RNA-seq is not from the strand-specific mRNA libraries. The editing site from the sense strand (+) was considered complementary to the antisense strand (-) because the transcriptome data containing antisense transcripts in this study, such as A-to-G + from the sense strand correspond to T-to-C- type from the antisense strand. And the statistics result showed the percentage of editing types was roughly equal between complementary editing types (Fig. 1a). Hence, we considered the complementary sites were sense strand editing types, and we finally identified 9 editing types. The statistics result showed that adenosine-to-guanosine (A-to-G+) was the main editing type which accounts for 95.99%, and the sums of other types were less than 5% (Fig. 1b). In addition, the statistical result showed the number of hyper editing sites was more than the number of regular editing sites in 15 skin tissues (Fig. 1c). In mammals, A-to-I editing was considered the most common editing type. To ensure the reliability of the editing sites in this study, we finally identified 54,707 A-to-I (G) editing sites for further study (Additional file 1). The editing ratio analysis showed a larger number of editing sites with a low editing ratio during the HFs cycle (Fig. 1d).

### Genomic distribution of A-to-I editing sites

To investigate the function of A-to-I edited genes, we analyzed the RNA structure location of editing sites. The results showed that A-to-I editing mainly occurred in intergenic, accounting for 65.31%, followed by introns, 5′UTR, 3′UTR, and coding sequence (CDS). We know RNA editing events in the protein-coding regions may alter the amino acid sequences and influence the protein functions, then, we further analyzed the editing events in the CDS region. Among these editing sites, 69.64% of editing events caused missense changes in protein sequence (Fig. 1e). A total of 117 A-to-I editing sites resulted in amino acid changes in 22 genes, including 7 known genes and 15 novel genes (Additional file 2).

### RNA editing events influence hair cycle-related genes by altering miRNA regulation

**Fig. 2:**
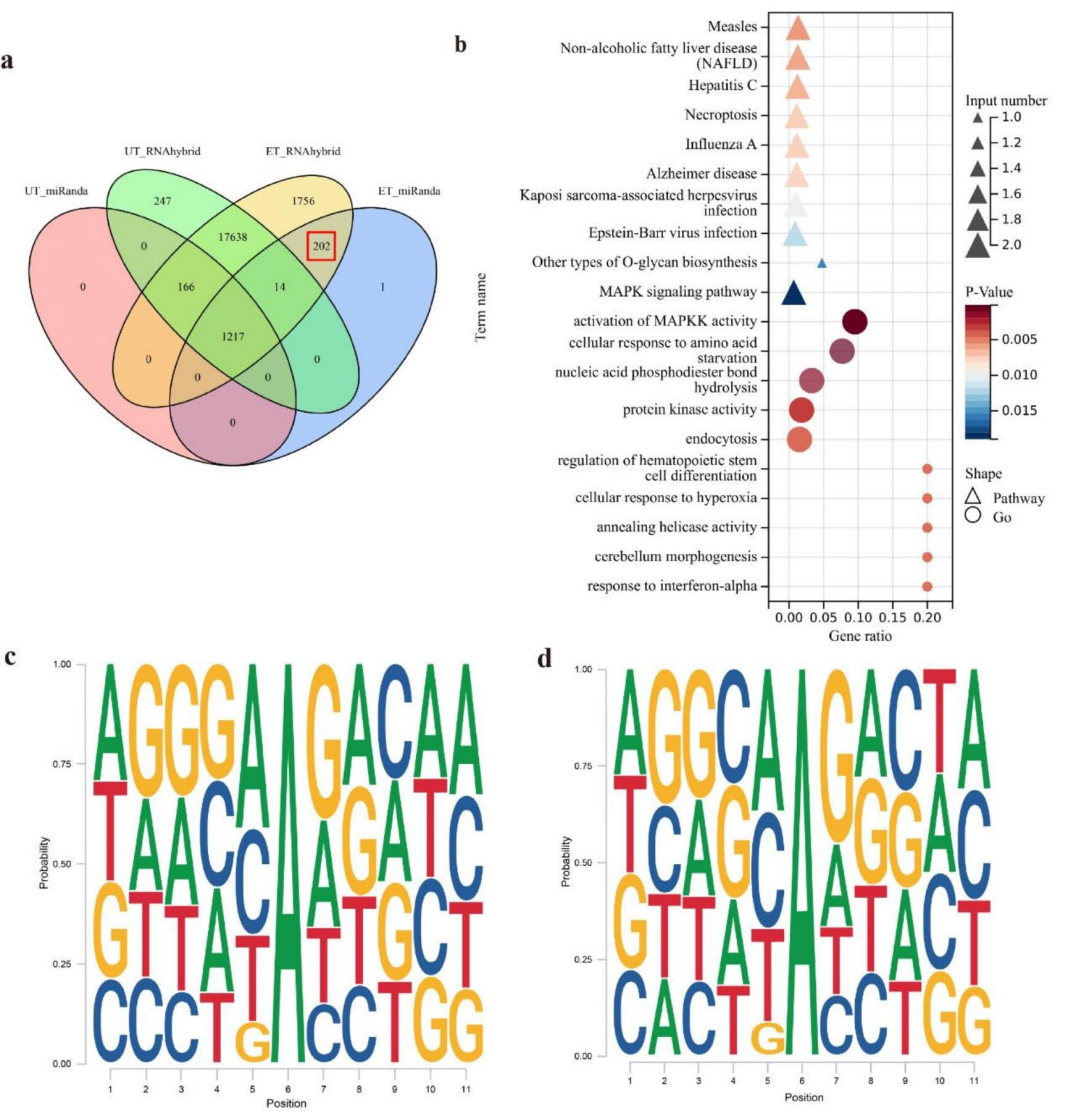
The influence of A-to-I editing events on miRNA binding genes and the sequence preference of A-to-I editing sites. (**a**) The Venn diagram of RNA editing altered miRNA binding sites, the intersection of UT represents loss target sites of miRNA; the intersection of ET represents the gain target sites of miRNA; (**b**) Top 10 pathways and top 10 GO terms of altered target genes of miRNAs; (**c**) Sequence preferences of the neighborhoods of hyper A-to-I editing sites; (**d**) Sequence preferences of the neighborhoods of regular A-to-I editing sites

Genome distribution analysis showed that 0.88% of editing sites occurred in 3′UTR (Fig. 1e). The edited 3′UTR may alter gene expression by changing the binding sites of the miRNA target sequence. Therefore, we analyzed the influence of the editing events on the relationship between the 3′UTR of genes and miRNAs. Based on the predicted miRNA-mRNA 3′UTR interactions, we defined 202 editing sites that caused a gain of miRNA binding targets (Fig. 2a, Additional file 3). Then we analyzed the relationship between miRNA and mRNA and selected the changed targets of miRNA for the functional enrichment analysis. All edited target sites were annotated to 23 genes. Kyoto encyclopedia of genes and genomes (KEGG) pathways and Gene Ontology (GO) analysis showed these targets were significantly enriched in the Necroptosis, MAPK, and activation of MAPKK activity terms, etc. (Fig. 2b)

### Conservation and sequence preference of A-to-I editing sites

Conservation analysis showed 101 editing sites were conserved between the human and yak, and only 2 editing sites were conserved between the mouse and yak (Additional file 4). There were no editing sites were conserved across the human, mouse, and yak. In addition, it has been found that the sequence preference of A-to-I editing sites is consistent with the ADAR sequence preference [31]. In this study, we respectively analyzed the sequence context flanking of identified regular and hyper A-to-I editing sites. We found both regular and hyper A-to-I editing sites with a low preference for G in the (− 1) base position and a high preference for G in the (+ 1) base position (Fig. 2c, d). The result is consistent with other studies [28, 32, 33].

### Differential analysis of A-to-I editing sites during hair follicle cycle of Tianzhu white yak

**Fig. 3:**
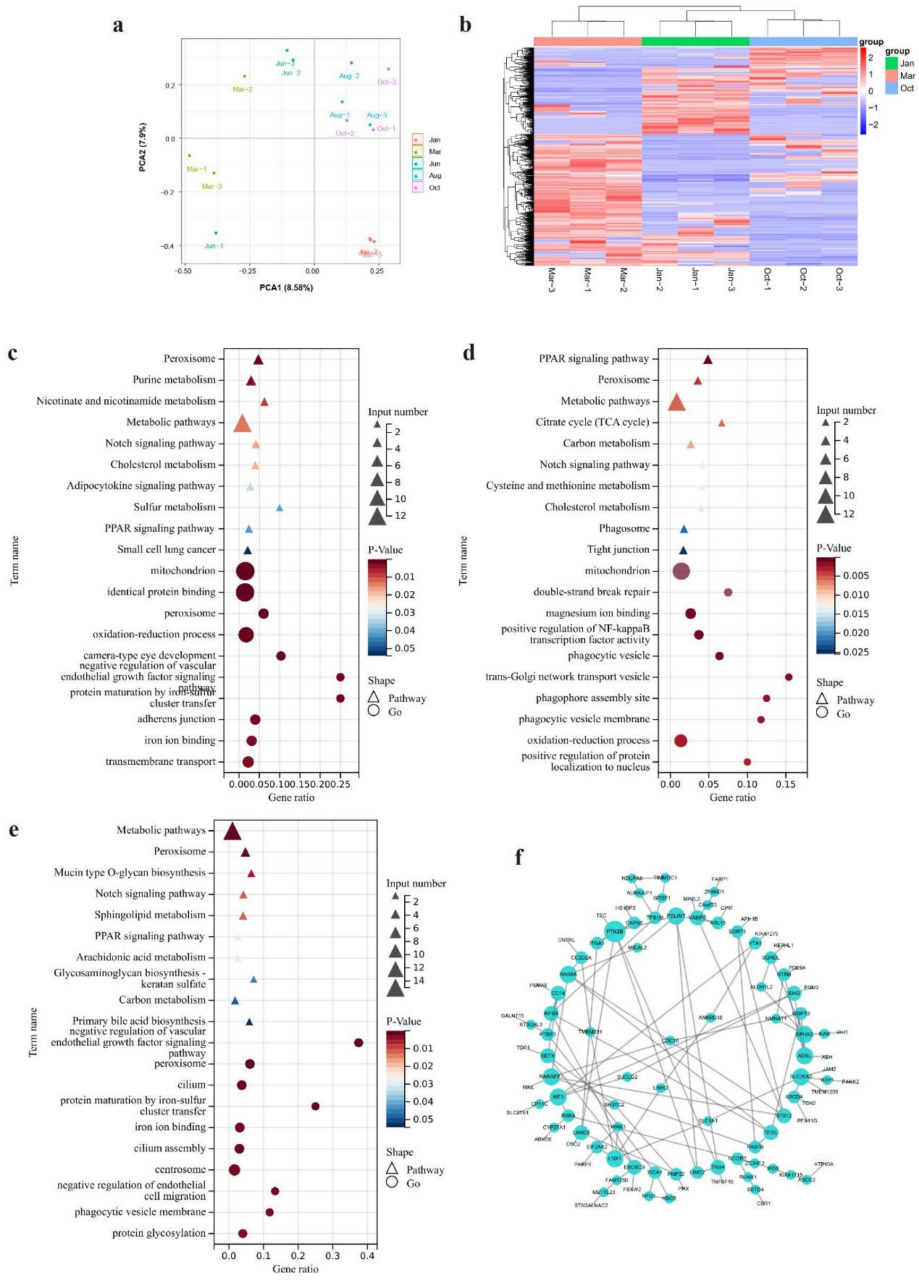
The differential RESs analysis and key RESs investigation. (**a**) PCA analysis of 15 skin samples; (**b**) Heatmap of the differential RESs; (**c**) Top 10 pathways and top 10 GO terms of the differential RESs edited genes (Jan vs. Mar); (**d**) Top 10 pathways and top 10 GO terms of the differential RESs edited genes (Jan vs. Oct); (**e**) Top 10 pathways and top 10 GO terms of the differential RESs edited genes (Mar vs. Oct); (**f**) The PPI network of all differential RESs between the different period of HFs cycle, the size indicates node degree

To further investigate how RNA editing events regulate the HFs cycle, we analyzed RESs with the differential editing levels between different periods of the HFs cycle. Firstly, we classified 15 skin samples into three groups according to the prior study. Then, we performed the principal component analysis (PCA) of the editing level to analyze sample similarities (Fig. 3a). According to the sample-to-sample distance in the PCA result, we finally selected 9 samples which were from Jan, Mar, and Oct groups for differential analysis. Besides, the samples from Jan, Mar, and Oct respectively represent telogen, catagen, and anagen according to previous report. The results showed that there have 898, 568, and 710 RESs were differential in Jan-vs-Mar, Jan-vs-Oct, and Mar-vs-Oct, respectively. Heatmap showing the differential RESs between different periods of HFs cycle. And the 9 samples were clustered into three significant groups in the heatmap (Fig. 3b). Then, we performed GO and KEGG enrichment analysis of differential RESs between different groups. The results revealed metabolic pathways, peroxisome, PPAR signaling pathway, and Notch signaling pathway were significantly enriched in Jan-vs-Mar, Jan-vs-Oct, and Mar-vs-Oct groups (Fig. 3c-e). The protein-protein interaction network showed PTK2B was a key node of the differential RESs edited genes (Fig. 3f).

### Validation result of identified A-to-I editing sites

To ensure the reliability of identified A-to-I editing events, we randomly selected two types of RESs (A-to-G + and T-to-C-) for validation. The sequence result showed that most nucleotide variations existed in the cDNA sequence but not in the corresponding position of the DNA sequence (additional file 5). The sequencing result verified the candidate A-to-I RESs in our study were reliable.

## Discussion

The HFs cycle is a complex process that is strictly regulated by genetics, environment, and nutrition. Among those factors, gene regulation plays a vital role in the periodic growth of HFs, and the RNA transcripts undergo a series of different processing mechanisms. Such RNA editing also can increase the diversity of transcripts by altering the nucleotide composition of transcripts. It would be interesting to explore the function of RNA editing in the HFs cycle. In this study, we firstly detected the RNA editing events during the HFs cycle of Tianzhu white yak and identified 56,994 RESs including 9 editing types. Same to other studies that A-to-I editing is the main type [26, 29, 34]. In mammals, A-to-I and C-to-U editing are the common types, and we generally regard other editing types as false-positive detections because of the limitations of current sequencing technology and analysis [30, 35]. In our study, A-to-I editing sites account for a large percentage of identified RESs. To ensure the accuracy of the identified sites, we finally selected A-to-I sites as the candidate RNA editing for further analysis.

The distribution of genomic location showed that 0.88% of A-to-I RNA editing events occurred in coding regions. Among those editing sites, 117 (69.64%) were missense variants, which cause amino acid sequences to change in 22 protein-coding genes including 7 known genes and 15 novel genes. The transcriptomes work of humans and mice has also identified thousands of RESs located in the CDS region [36]. Although we found only a few protein-coding genes may be influenced by RESs, we know protein changes could directly influence biological function. The seasonal HFs cycle is important for a yak to adapt to an extreme temperature in the plateau area. The heat shock proteins with a vital role in livestock adaptation to cold stress. In this study, we found the amino acids of heat shock protein-coding genes *HSF2BP* and *HSPA1L* were influenced by RNA editing. It has been verified that the genetic variation in the promoter region of heat shock protein HSPA1L could protect cells from elevated body temperature [37]. A previous study verified that overexpressed *Dusp6* promoted the expression of *Hspa1l* meanwhile inhibiting the proliferation of hair follicle stem cells [38]. In this study, the amino acid of HSPA1L was affected by RESs. We speculate that *HSPA1L* may play an important role for a yak to adapt to the plateau climate by increasing cell survival and regulating the seasonal cycle of the HFs. MiRNA act as a key post-transcriptional regulator of gene expression, which has been reported with important roles in the HFs cycle. A-to-I editing can alter gene expression by changing the binding sites within the 3′UTR of the miRNA target sequence. In our study, RNA editing altered 23 target genes of 140 microRNAs. We found these genes were enriched in the MAPK pathway and activation of MAPKK activity GO term. Previous studies reported that MAPK signaling participates in the hair cycle and the quiescence of HF stem cells [8, 39]. In addition, we found that RNA editing also changed the target sites of FAS. It was reported that FAS-deficient mice avoided hair loss when induced alopecia areata [40]. Kim’s study also showed FAS/FAS ligand pathway mediated apoptosis-regression catagen in HFs [41]. Hence, we speculate that RESs may regulate the HFs cycle by altering the target sites of miRNA.

In the previous study, the RNA editing ratio was dynamically changed to respond to the different biological situations or stages. In this study, we analyzed the RESs with dynamically changed editing levels during the HFs cycle. Enrichment analysis results of differential RESs showed that three study groups were all enriched in the peroxisome, metabolic pathways, notch signaling pathway, and PPAR signaling pathway. And these signaling pathways were reported to be essential for HFs development. For example, Notch signaling determines the HFs stem cell fate [42]. And Peroxisome proliferator-activated receptors (PPARs) were investigated in skin tissue, its subtypes PPARα and PPARβ could participate in skin homeostasis and normal hair follicle morphogenesis [43]. In addition, we found some differential RESs edited genes function in skin development, hair growth, and HFs cycle have been reported, such as *APCDD1*, *WWOX*, *MPZL3*, *RUNX1*, *KANK2*, *DCN*, *DSC2*, *LEPR*, and *HEPHL1*. Previous studies revealed that the mutation of *APCDD1* was associated with hair loss in hereditary hypotrichosis simplex patient, and suggested *APCDD1* as an inhibitor of WNT pathway may cause HFs miniaturization and hair loss [44]. In the sheep, *APCDD1* was also considered a potential factor that caused the various wool characteristics [45]. The subcutaneous fat might improve the tolerance of livestock to extremely cold environments. A previous study showed that more than 95% of the subcutaneous fat was lost in *Wwox*-/- mice. And the HFs stem cell numbers were also reduced in *Wwox*-/- mice. Cao et al. revealed the rough coat mice have a missense mutation in the *Mpzl3* gene [46]. The normal growth of HFs is indispensable for skin development and homeostasis. It has been found the abnormal function of *Mpzl3* involves a skin disorder. The *Mpzl3*-/- mouse appeared with sebaceous hypertrophy and hair loss soon after birth [47]. In addition, it has been described that *Mpzl3* is involved in epidermal differentiation by interacting with mitochondrial protein [48]. Nicu et al. provided an insight that *Mpzl3* controls HFs cycling may be related to mitochondria protein activity [49]. Runx1 is a transcription factor was associated with the HFs cycle. It has been found that *Runx1* could promote the proliferation/activation of HFs stem cells and regulate HFs cells from telogen to anagen transition [50, 51]. Lee et al. found the expression of endogenous *Runx1* was associated with the differentiation and dedifferentiation of HFs bulge stem cells and maintained a balance between HFs stem cells and early progenitor populations during normal HFs catagen [52]. A previous sequencing result revealed that human with woolly hair has a homozygous missense mutation in *KANK2* [53]. HFs stem cells decide hair regrowth and its maintenance relies on the specific niche. It is known the number of HFs stem cells decreases with age, as HFs stem cell niche component, *DCN* has been suggested as a candidate factor that is related to age-regulated hair loss [54]. The development of epithelial cells is essential for hair morphogenesis, Desmocollin-2 (*DSC2*) has been demonstrated plays a vital role in regulating epithelial morphogenesis [55]. The expression of *Dsc2* reduction could lead to defective hair morphogenesis in nude mice [56]. In goats, *DSC2* is also the candidate factor related to woolly or straight hair [57]. Leptin (Lep) participates in HFs morphogenesis and cycle [58]. *LEP* and its receptor (*LEPR*) were observed expressed in hair follicles of bovine skin [59]. It has been revealed that *Lepr* expressed in dermal condensates during HFs morphogenesis, and *Lepr* could as a marker for dermal papilla cell isolation [60]. *HEPHL1* is a member of the multicopper oxidase family, it has been reported that two mutations in *HEPHL1* caused abnormal hair phenotype, and the Hephl1 knockout mice appeared with curly whiskers [61]. The protein-protein interaction result revealed that *PTK2B* was a hub gene among differential RESs edited genes. The normal growth of hair is closely related to the development of skin. Koppel et al. revealed that function loss of *Ptk2b* caused wound repair delays in mice [62]. All evidence suggested that RESs may involve the HFs cycle by regulating the HFs growth-related genes.

## Conclusion

In conclusion, this study detected the amount of A-to-I editing events of Tianzhu white yak during the HFs cycle using RNA-Seq data, and systematically analyzed the protein-coding region, interaction relation between miRNA and mRNA, and the differential RESs during the HFs cycle. GO and KEGG enrichment results of genes with dynamically changed editing level during the HFs cycle suggested several signaling pathways related to skin development, hair growth, and HFs cycling. The findings of this study might provide new insight into the study of RNA editing regulation and mechanism in hair growth and HFs cycle.

## Methods and Materials

### The description of RNA-seq datasets and reads mapping

To investigate the RNA editing events during the HFs cycle of Tianzhu white yak, we retrieved 15 publicly available paired-end RNA-Seq data. The data were generated from the skin tissue of Tianzhu white yak from three stages of the HFs cycle containing five-time points (January, March, June, August, and October), and each time point has three replicates. (NCBI, accession number PRJNA550233). The detailed information of samples was shown in (Additional file 6). As the previous study described, five stages could be divided into three development periods of HFs cycle [14]. The RNA libraries were generated using the Illumina HiSeq 2500 platform. For each library, clean reads were obtained after removing reads containing adapters, reads containing poly-N sequences, and low-quality reads using SOAPnuke (v2.1.0) software [63]. The RNA-seq clean reads were aligned to the *Bos grunniens* reference genome (LU_Bosgru_v3.0) and used BWA with default parameters. Subsequently, the sequence alignment/map format (sam) files were sorted and converted to BAM files by Samtools (1.9).

### RNA editing site detection and annotation

The editing sites were detected by the SPRINT software (https://github.com/jumphone/SPRINT) using the default parameters. The SPRINT software could identify RNA editing events without the need to filter out single nucleotide polymorphism (SNPs) according to the difference in distribution characteristics between RESs and SNPs [64]. To ensure the accuracy of identified RESs. We retained the editing sites at least supported by three samples We used the snpEff (4.3t) software to annotate RESs [65]. While the LU_Bosgru_v3.0 version reference genome download from Ensemble datasets was used for annotation. Based on referential gene data, we can analyze the RNA editing locate in which genes, the distribution of editing sites on the genome, and which RESs can cause protein mutations.

### Analysis of the RNA editing effects on miRNA regulation

To investigate the impact of RNA editing on miRNA-mRNA binding regions, we used MiRanda (3.3a) and RNAhybrid (2.1.2) to predict miRNA-mRNA 3′UTR interactions. Based on the unedited reference targets regions (3′UTR) of miRNAs as control. Edited sequence caused new interaction of miRNA was defined as the gain of miRNA binding targets supported by two software, on the contrary, the loss of miRNA binding targets in edited sequence caused referential interaction of miRNA binding targets loss. Then, the altered target genes of KEGG and GO were enriched by KOBAS 3.0 (http://kobas.cbi.pku.edu.cn/anno_iden.php).

### Conservation and sequence preferences analysis of A-to-I editing

To analyze the conservation of editing sites across the yak, humans, and mice. We aligned the 25- base sequence upstream and downstream of A-to-I editing sites against 50 bp flanking regions of the reported human sites by using the BLASTn tools. Human and mouse RNA editing site data were downloaded from REDIportal (http://srv00.recas.ba.infn.it/atlas/) databases [36]. We deemed alignments with e-values < 1e-5 and more than 85% identity were considered as conserved editing sites. Additionally, we extracted 5-base sequences upstream and downstream of the A-to-I editing sites to investigate the flanking sequence preferences.

### Differential RNA editing analysis

The dynamically changed editing levels during the HFs cycle may with an important function. A-I editing level was quantified as the ratio of the number of G (C) reads to the total number of A (T) and G (C) reads covering the site. The method of editing-level computing was described by Cai. An unpaired student’s t-test method was used for the differential RNA-editing sites analysis (P-value < 0.01) between different periods of the HFs cycle. Then, the KEGG and GO results of differential RESs annotated genes were enriched by KOBAS 3.0. GO terms and KEGG pathways were defined as significant with Fisher’s exact test P-value < 0.05. Then, we used the online STRING database (https://cn.string-db.org/) and Cytoscape_3.7.2 software to construct the interaction network of the differential RESs edited genes-corresponding proteins.

### The validation experiment of identified RNA editing sites

To ensure the reliability of the identified RNA editing events by SPRINT software. We used PCR and sanger sequence methods to validate the randomly selected A-G and T-C types. The animals (2 ~ 3 years old healthy female yak) for the validation experiment were collected from the Tianzhu Tibetan Autonomous County, Gansu Province of China at Jun (Telogen). A total of 19 skin samples and 19 venous blood samples were collected from the anesthetized yaks. The blood and skin samples were collected from the same individual. The operation of sample collection was allowed by the herdsman. We used the skin biopsy punches (8-mm-diameter) to collect skin samples of yak after injecting a local anesthetic (2% lidocaine) into the subcutaneous of the shoulder. We had made all efforts to minimize the suffering of the animals studied here. The animal study is approved by The Animal Administration and Ethics Committee of Lanzhou Institute of Husbandry and Pharmaceutical Sciences of CAAS (Permit No. SYXK-2014-0002). The study is in accordance with all the guidelines and regulations. The study is in accordance with the ARRIVE Guidelines. Total RNA of skins tissue was extracted using TRIzol reagent (Invitrogen, CA, USA), and then the total RNA was used as templates for cDNA generation by TransScript® One-Step gDNA Removal and cDNA Synthesis SuperMix (TransGen Biotech, Beijing, China). The· blood genomic DNA was extracted using the BloodZol kit (TransGen Biotech, Beijing, China). PCR reactions were performed using GoTaq (Promega), gDNA was amplified by the mixed template gDNA, and cDNA was amplified by the mixed template cDNA. The primer and RESs information of amplification was shown in (Additional file 7). All experiment steps were following the manufacturer’s protocol.

## Electronic supplementary material

Below is the link to the electronic supplementary material.

Additional file 1: Identified A-to-I editing sites of hair follicles cycle in Tianzhu white yak

Additional file 2: The information of protein-coding genes with missense RNA editing sites

Additional file 3: The influence of RNA editing on the relationship between miRNA and mRNA

Additional file 4: The information of conserved RNA editing sites

Additional file 5: The validation result of RNA editing sites

Additional file 6: The information of 15 skin samples

Additional file 7: The information of the primer and validated RNA editing sites

## List of abbreviations

HFs: hair follicles
RNA-Seq: RNA sequencing
RESs: RNA editing sites
SNPs: single nucleotide polymorphism
CDS: coding sequence
KEGG: Kyoto encyclopedia of genes and genomes
A-to-I (G): adenosine-to-inosine
MiRNA: microRNA.
