# Supplementary figures and images for: Genome-wide detection of RNA editing events during the hair follicles cycle of Tianzhu white yak
Source: BMC Genomics. 2022 Oct 31;23:737. doi: 10.1186/s12864-022-08951-5 (PMC9624038; doi:10.1186/s12864-022-08951-5)

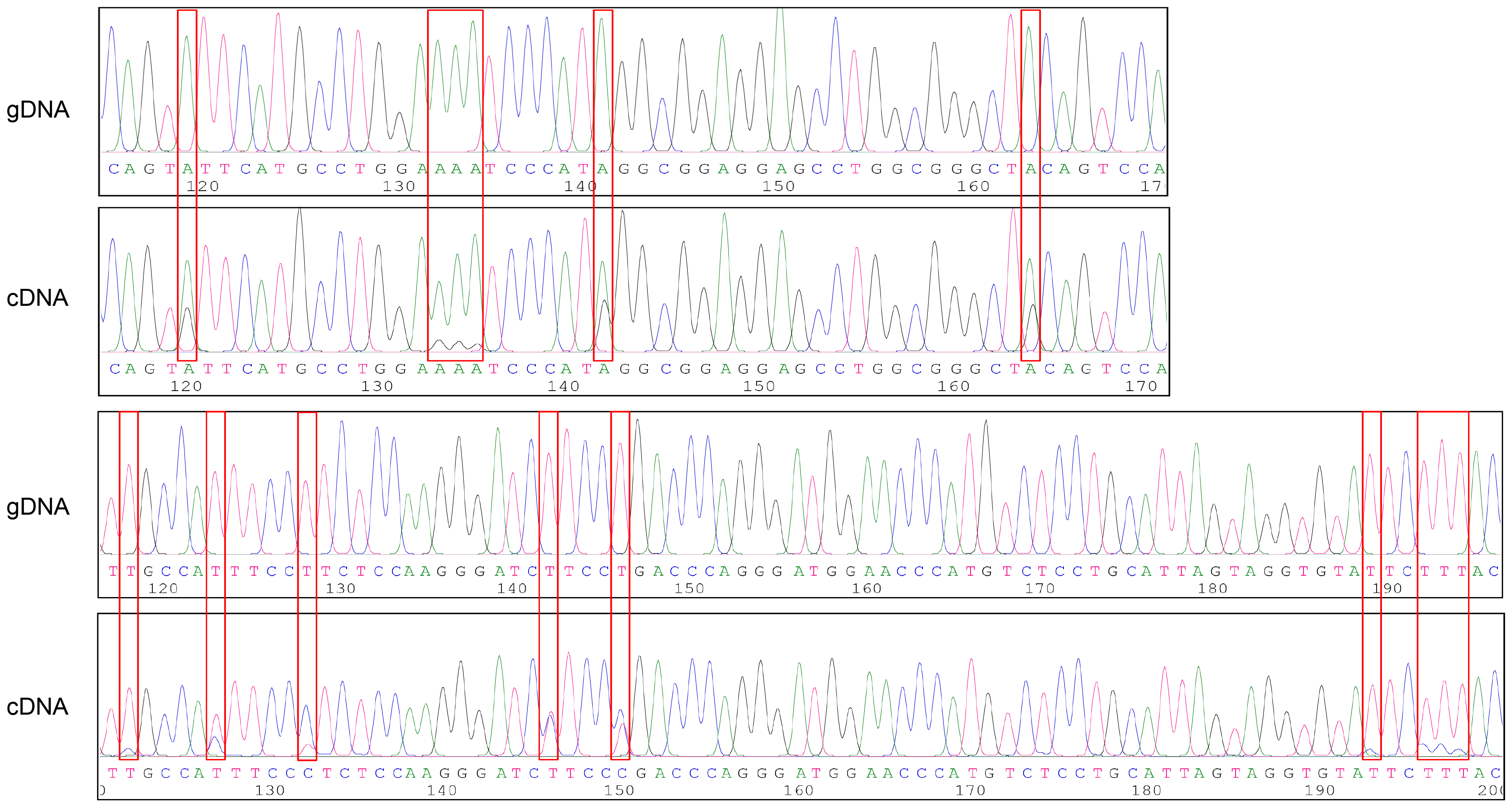

Supplement: Supplementary file 5 — Additional file 5: The validation result of RNA editing sites [file 12864_2022_8951_MOESM5_ESM.tif]
