# Supplementary material for: Genome-wide detection of RNA editing events during the hair follicles cycle of Tianzhu white yak
Source: BMC Genomics. 2022 Oct 31;23:737. doi: 10.1186/s12864-022-08951-5 (PMC9624038; doi:10.1186/s12864-022-08951-5)
Supplement: Supplementary file 6 — Additional file 6: The information of 15 skin samples [file 12864_2022_8951_MOESM6_ESM.docx]

Table S1 The information of 15 skin samples

| Sample Name | Accession | Period |
| --- | --- | --- |
| Jan-1 | SRR10059246 | Catagen |
| Jan-2 | SRR10059245 | Catagen |
| Jan-2 | SRR10059239 | Catagen |
| Mar-1 | SRR10059238 | Telogen |
| Mar-2 | SRR10059237 | Telogen |
| Mar-3 | SRR10059236 | Telogen |
| Jun-1 | SRR10059235 | Telogen |
| Jun-2 | SRR10059234 | Telogen |
| Jun-3 | SRR10059233 | Telogen |
| Aug-1 | SRR10059232 | Anagen |
| Aug-2 | SRR10059244 | Anagen |
| Aug-3 | SRR10059243 | Anagen |
| Oct-1 | SRR10059242 | Anagen |
| Oct-2 | SRR10059241 | Anagen |
| Oct-3 | SRR10059240 | Anagen |
