# Supplementary material for: Genome-wide detection of RNA editing events during the hair follicles cycle of Tianzhu white yak
Source: BMC Genomics. 2022 Oct 31;23:737. doi: 10.1186/s12864-022-08951-5 (PMC9624038; doi:10.1186/s12864-022-08951-5)
Supplement: Supplementary file 7 — Additional file 7: The information of the primer and validated RNA editing sites [file 12864_2022_8951_MOESM7_ESM.docx]

Table S2 The information of the primer and validated RNA editing sites

| primer | Amplification region | RNA editing site of validation |
| --- | --- | --- |
| Primer 1  F: GCTGCAGATGCCAGAAACGAG  R: AGAAGGGATGGGTAACTGTGATG | 1:36967830-36968126 | 1_36967980_36967981_AG_+ |
|  |  | 1:36967993_36967994_AG_+ |
|  |  | 1:36967994_36967995_AG_+ |
|  |  | 1:36967995_36967996_AG_+ |
|  |  | 1:36968002_36968003_AG_+ |
|  |  | 1:36968024_36968025_AG_+ |
| Primer 2  F: AAAGAACCACTTCCGTGCC  R: GGGAAGTGGGGCAAATTATGTT | 11:76993884-76994246 | 11:76994034_76994035_TC_- |
|  |  | 11:76994039_76994040_TC_- |
|  |  | 11:76994044_76994045_TC_- |
|  |  | 11:76994058_76994059_TC_- |
|  |  | 11:76994062_76994063_TC_- |
|  |  | 11:76994105_76994106_TC_- |
|  |  | 11:76994108_76994109_TC_- |
|  |  | 11:76994109_76994110_TC_- |
|  |  | 11:76994110_76994111_TC_- |
